# Comparative study of extraction methods of silver species from faeces of animals fed with silver-based nanomaterials

**DOI:** 10.1007/s00604-023-05777-0

**Published:** 2023-05-09

**Authors:** María S. Jiménez, Mariam Bakir, Khaoula Ben-Jeddou, Eduardo Bolea, Josefina Pérez-Arantegui, Francisco Laborda

**Affiliations:** Group of Analytical Spectroscopy and Sensors (GEAS), Institute of Environmental Sciences (IUCA), University of Zaragoza, Pedro Cerbuna 12, 50009 Zaragoza, Spain

**Keywords:** Silver fractionation, Nanomaterials, Liquid extraction, Single-particle ICP-MS, Hydrodynamic chromatography

## Abstract

Extractions methods based on ultrapure water, tetramethylammonium hydroxide (TMAH), and tetrasodium pyrophosphate (TSPP) were applied to faeces collected from two in vivo experiments of pigs and chickens fed with a silver-based nanomaterial to study the fate and speciation of silver. For TMAH extraction, cysteine and CaCl_2_ were used to evaluate their stabilization effect on the silver forms. The analytical techniques single-particle inductively coupled plasma mass spectrometry (SP-ICP-MS), hydrodynamic chromatography hyphenated to ICP-MS (HDC-ICP-MS) and asymmetric flow field flow fractionation coupled to ICP-MS (AF4-ICP-MS) were applied to the simultaneous detection of particulate and dissolved silver. Results have shown that water extraction was a suitable option to assess the environmental release of silver, with percentages of 3 and 9% for faeces of pigs and chickens, respectively. The use of TMAH extraction combined with SP-ICP-MS analysis was useful to characterize Ag-containing particles (less than 1%). Both stabilizers, cysteine and CaCl_2_, have a similar effect on silver nanoparticle preservation for chicken faeces, whereas cysteine-Triton was better for pig samples. In any case, silver extraction efficiency with TMAH was low (39–42%) for both types of faeces due to a matrix effect. TSPP followed by ICP-MS enabled the fractionation of the silver in the faeces, with silver sulphide (41%) and ionic silver (62%) being the most abundant fractions.

**Graphical Abstract:**

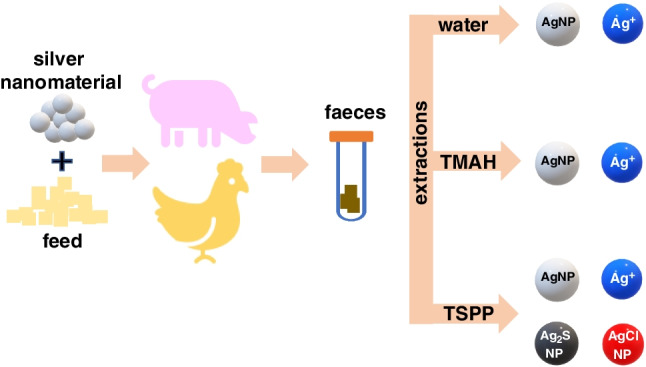

**Supplementary Information:**

The online version contains supplementary material available at 10.1007/s00604-023-05777-0.

## Introduction

The increase in the use of engineered nanomaterials (ENMs) and their release into the environment makes necessary the evaluation of their potential risks. Silver nanoparticles (AgNPs) are widely used for their antibacterial properties [1]. They are incorporated in several products like medical and cosmetics products, food additives, textile coatings or food packaging [2, 3]. AgNPs are also studied as feed additives for animals [4, 5] with the aim of substitution of antibiotics. The elimination of the consumed AgNPs through the faeces can be one of the sources of the release into the environment. Information about the silver forms in the excreta of animals fed with nano-additives is part of the recommended risk assessment of nanomaterials in the guidance of the European Food Safety Authority [6]. The toxicity of AgNPs depends on the particle properties (size, form, concentration) [7]. Subsequently, a suitable method for NPs characterization is essential for the risk assessment of nanomaterials, but, until the moment, there is no standard method for the characterization and quantification of NPs in faeces due to the complexity of the nature of excrements making the determination of silver species challenging.

Several sample preparation methods have been studied to digest biological samples containing NPs [8]. Acid digestion is frequently used to digest the nanoparticle-containing tissues [9, 10]. However, the strong acids dissolve the nanoparticles; thus, other extraction methods that enable the conservation of the integrity of nanoparticles are needed.

For most of the biological samples, alkaline extraction with TMAH [11–16] and enzyme-based extraction [17–21] are commonly used. TMAH is a strong base able to break organic and biological matrices [22]. To avoid changes in the NP morphology, a stabilizer is needed. Clark et al. [23] demonstrated that the use of TMAH alone caused the precipitation of Ag(I) in spiked fish, which was also demonstrated by Vidmar et al. [18] in a human placenta. Therefore, CaCl_2_ was proposed as a stabilizer as it avoided Ag(I) precipitation. Jiménez-Lamana et al. [15] used cysteine to stabilize Ag(I) and Triton X-100 as a surfactant to stabilize AgNPs. The use of surfactants was reported in other studies [22, 24, 25] to prevent the loss of NPs and to recover the total spiked Ag. Gao et al. [24] also studied the process of sample grinding and concluded that it improved NP extraction leading to recoveries of nearly 100% for gold nanoparticles in mice organs. Enzymatic extraction is used for its ability to extract NPs without alterations from the tissues [21]. Generally, enzymatic extraction is conducted using proteinase K [26], but this enzyme can partially dissolve AgNPs, and the presence of matrix residues leads to a lower nanoparticle recovery [17]. Comparisons between alkaline and enzymatic extractions were reported [25, 27], obtaining better results with the alkaline extraction. Sung et al. [27] observed that TMAH gave higher recoveries (102%) than enzymatic extraction (74%) for the extraction of AgNP and AuNP from zebrafish liver samples.

Tetrasodium pyrophosphate (TSPP) has been frequently used for the extraction and fractionation of the different element species with special attention to particulate forms [28–30]. Hong et al. [30] developed a sequential extraction for different species of Ag (Ag(I), AgNPs, AgCl and Ag_2_S) in spiked soils based on TSPP and sodium sulphide (Na_2_S) measuring the resultant solutions by inductively coupled plasma optical emission spectrometry (ICP-OES). This method provided useful information about the nature of the Ag species in the spiked samples and their proportion. However, the use of aqueous ammonium and Na_2_S as extractants caused a dissolution and complexation of Ag_2_S and AgCl, leading to a loss of information about the size and morphology of the particles.

Water extraction is used to study the fate and transformations (e.g., dissolution, oxidation, aggregation) of NPs during the release process [31]. Animal manure is used as organic amendment to improve soil fertility [32]. However, these amendments can be a sink for metal accumulation, being important to evaluate their mobilization into the environment using water extraction protocols [33]. Water extraction followed by AF4 analysis was used for the size characterization of nanocolloids, macromolecules and microparticles in compost [34]. The authors observed different mobilization behaviours of several metals depending on their affinity of association for the particulate or macromolecule fractions. Water extraction of soils containing faeces of earthworms fed with cobalt NPs, AgNPs and Ag (I) revealed a silver concentration below the detection limit [35].

The objective of this study is to evaluate the efficiency of three different strategies, based on the use of water, TMAH and TSPP, for the extraction of dissolved and nanoparticulate silver species from faeces of pigs and chickens that were orally fed with a silver-based nanomaterial. The behaviour of the silver species along the three extractions protocols will be studied by using different ICP-MS-based techniques (SP-ICP-MS, HDC-ICP-MS and AF4-ICP-MS).

## Materials and methods

### Chemicals

Aqueous gold solutions were prepared from a standard stock solution of 999 ± 4 mg L^−1^ (Panreac, Barcelona, Spain) by dilution in ultrapure water (Milli-Q Advantage, Molsheim, France) by accurately weighing (± 0.1 mg). Aqueous silver (I) (Ag(I)) solutions were prepared from a standard stock solution of 994 ± 3 mg L^−1^ (Panreac, Barcelona, Spain) by dilution in ultrapure water. Diluted suspensions of AgNPs were prepared from commercial suspensions of AgNPs with sizes 10.3 ± 2.1, 19.1 ± 3.6, 39 ± 5 and 59 ± 6 nm (Nanocomposix, San Diego, USA). AuNPs of 47.8 ± 1.8 nm (Nanocomposix, San Diego, USA) with a concentration of 52 mg L^−1^ were used as a certificated material of size and concentration.

For the mobile phase used in HDC separation, a solution of 0.45 mM sodium dodecyl sulphate (SDS) (Bio-Rad Laboratories, California, USA, electrophoresis purity reagent, ≥ 98.5%) and 1 mM D-Penicillamine (PA) (Sigma Aldrich, St. Louis, USA, 98–101%) in 1 L of ultrapure water was prepared. To reach the pH 7.5 necessary for the measurements, a sodium hydroxide solution (Prolabo, Fontenay-Sous-Bois, France, 98%) and a nitric acid solution (J.T. Baker, Phillipsburg, USA, 69/70%) were used.

The extracting reagents used were TMAH 25% (Alfa Aesar, Kandelm Germany, electronic grade, 99.9999 %), TSPP (Sigma-Aldrich, Sta. Louis, USA, ≥ 95%) and Na_2_S (Sigma-Aldrich, Sta. Louis, USA, ≥ 99.99% trace metals basis). Cysteine (Sigma-Aldrich, Sta. Louis, USA, BioUltra, ≥ 99.0%.), Triton X-100 (Sigma-Aldrich, Sta. Louis, USA, laboratory grade), CaCl_2_ (Fisher Scientific, Loughborough, UK, analytical reagent grade) and aqueous ammonium (NH_3_) (30%, J.T.Baker, Phillipsburg, USA, ≥ 99.5% trace metals analysis) were also used.

### Instrumentation

ICP-MS in conventional mode and SP-ICP-MS analysis were conducted using a Perkin Elmer model NexION 2000 ICP mass spectrometer (Toronto, Canada). The sample introduction system consisted of a baffled cyclonic spray chamber and a concentric nebulizer (Meinhard, Colorado, USA). The isotopes monitored were ^107^Ag and ^109^Ag. Syngistix™ 2.5 (Perkin Elmer, Toronto, Canada) software, and the nano module for the SP-ICP-MS measurements were used for the acquisition of the data. The experimental conditions are described in table T1 in supporting info (SI). Before all the analysis, ICP-MS measurement conditions were optimized according to the manufacturer instructions, and to achieve the maximum sensitivity for Ag, nebulizer gas flow and lens voltage were optimized using a 1 μg L^−1^ Ag standard. The transport efficiency, required for SP-ICP-MS calculations, was determined by the size method developed by Pace et al. [36] which relies on a calibration with ionic Au standards and the use of a Au NP size standard (50 nm).

For HDC-ICP-MS analysis, the high-performance chromatographic system used was a Waters 2796 Bioseparations module (Waters Corporation, Milford, USA). HDC separations were performed with a PL-PSDA Type 1 column (Agilent Technologies, Germany) with a nominal separation range of 5–300 nm, a length of 80 cm and an internal diameter of 7.5 mm. The sample flow rate used was 1.6 mL min^−1^, and the mobile phase used was 0.45 mM of SDS and 1 mM of PA, optimized in previous studies [37]. It was coupled to a Perkin Elmer Sciex model ELAN DRC-e ICP mass spectrometer (Toronto, Canada) and used as a detector. The outflow of the column was delivered directly to the nebulizer of the spectrometer which is a glass concentric slurry nebulizer with a cyclonic spray chamber (Glass Expansion, Melbourne, Australia). Before the analysis, ICP-MS measurement conditions were optimized to achieve the maximum sensitivity for Ag, using a 10 μg L^−1^ Ag (I) solution. The experimental conditions of the HDC and the ICP-MS are shown in table T1 in SI. The quantification of the peaks of the chromatograms is reported by Jiménez et al. [37].

For AF4-ICP-MS analysis, an AF2000 (Postnova Analytics, Landsberg, Germany) system was used. The trapezoidal channel was 14 cm in length and 2 cm in width, and the spacer used was 350 μm thick (Postnova Analytics, Landsberg, Germany). As an accumulation wall, an ultrafiltration membrane of polyether sulfone (PES) (cut-off 5 kDa; Postnova Analytics, Landsberg, Germany) was utilized. The mobile phase was ultrapure water, previously filtered. The crossflow programs are listed in table T2 in SI. The AF4 system was coupled to a Perkin Elmer Sciex model ELAN DRC-e ICP mass spectrometer, used as a detector. The outflow of the channel was delivered directly to the nebulizer of the spectrometer which is a glass concentric slurry nebulizer with a cyclonic spray chamber. Before the analysis, ICP-MS measurement conditions were optimized to achieve the maximum sensitivity for Ag, using a solution with a concentration of 20 μg L^−1^. The experimental conditions of the AF4 and the ICP-MS are shown in table T1 in SI.

For transmission electron microscopy (TEM) analysis, a Tecnai F30 (FEI Company, Eindhoven, Netherlands) equipped with an electron source with a working voltage between 200 and 300 kV was used. It was coupled to an energy-dispersive spectrometer (EDS). Twenty microliters of the sample digested (without no cleaning step prior the measurement) was deposited on a copper grid for the water extraction and on a gold grid for the alkaline extraction, and coated with carbon, once the sample was dry. To process the images obtained, ImageJ 1.53 version software was utilized.

Origin 9.6.5.169 (OriginLab, Northampton, MA, USA) was used for the processing of the data.

### Samples

Pig and chicken faeces were collected from an in vivo experiment in which the animals were fed with feed containing the silver-kaolin. Before sample treatment, lyophilized faeces were ground with a Restch MM400 mill (Restch, Düsseldorf, Germany). The grinding was done in stainless steel jars with a 25-mm ball at a frequency of 25 Hz for 3 min. The total Ag content was obtained by acid digestion and ICP-MS determination. The detailed results will be published. Mean total concentrations of silver in faeces obtained were 155 ± 30 and 1026 ± 178 mg kg^−1^ for pigs fed with silver-kaolin at a silver concentration of 20 and 200 mg kg^−1^, respectively, and an amount of Ag of 75 ± 12 mg kg^−1^ in faeces of chickens fed with 20 mg kg^−1^ of silver.

### Procedures

#### Water extraction

One hundred milligrams of ground faeces was weighed (± 0.1 mg) and added to 1 mL of ultrapure water, to obtain a solid/reagent ratio of 1:10 as described by Kosson et al. [38]. The suspensions were shaken at 28 rpm for 48 h at room temperature and darkness. Afterwards, the suspensions were sonicated for 5 min and left for 20 min at room temperature. Then, the samples were centrifugated at 7000 g for 5 min at 21 °C to deposit the particles larger than 1 μm in the bottom of the tube.

#### Alkaline extraction

For the alkaline extraction, the protocol proposed by Jiménez-Lamana et al. [15] was used. One hundred milligrams of ground faeces was put into 15-mL polypropylene tubes with 2 mL of TMAH 25% (w/w) and 0.4 mL of cysteine 0.5% (w/w). They were mixed in a tumbler for 24 h at 28 rpm at room temperature and in darkness. After that, the solutions were sonicated and made up to 10 mL with a solution of cysteine 0.1% and Triton X-100 0.05%. Two different separation modes were studied to investigate the loss of Ag due to the centrifugation process. After the alkaline extraction, the centrifugation (at 7000 g for 17 min at 21 °C) and the sedimentation were compared as separation methods. The sedimentation was done leaving the samples for 1 h in an ice bath to the deposition of particles larger than 1 μm in the bottom. Samples were diluted prior to their analysis.

#### TSPP sequential extraction

For the sequential extraction process, the protocol developed by Hong et al. [30] was employed. Five hundred milligrams of ground faeces was added to 8 mL of 10 mM TSPP, and the pH was adjusted with a solution of 2 mM of NaOH. Samples were shaken at 200 rpm and left in an ultrasound bath for 30 min. The samples were centrifugated for 10 min at 157 g. The supernatant was separated from the solid. The same procedure was carried out on this pellet again: 8 mL of the solution of TSPP, agitation, ultrasonication and centrifugation. Once it was centrifuged, the solid was separated from the supernatant, and the latter was combined with the first supernatant that had been extracted. The same procedure was performed with the remaining pellet, but, in this third case, only 4 mL of 10 mM TSPP was added. In total, the resulting supernatant was around 18 mL. This supernatant was divided into 3 subsamples: direct determination through ICP-MS representing the Ag(I), AgNPs and AgCl-NPs; centrifugation and determination by ICP-MS, representing the Ag(I) and adding NH_3_, centrifugation and determination by ICP-MS representing the Ag(I) and AgCl-NPs.

The resulting pellet was washed 3 times with ultrapure water, and 8 mL of 0.1 M of Na_2_S was added. The procedure that was followed is the same as that followed with the TSPP, and the suspension was shaken, ultrasonicated and centrifuged. The process was repeated two more times (with 8 mL and 4 mL more of Na_2_S), obtaining approximately 18 mL of supernatant. With this step of fractionation, the Ag_2_S-NP fraction was extracted.

## Results and discussion

### Study of the different extraction methods for the extraction of Ag species from faeces

Ultrapure water, alkaline extraction with TMAH and sequential extraction with TSPP and Na_2_S were evaluated as extraction reagents of silver forms from faeces of pigs and chickens fed with feed containing a silver-kaolin nanomaterial.

### Water extraction

**Fig. 1:**
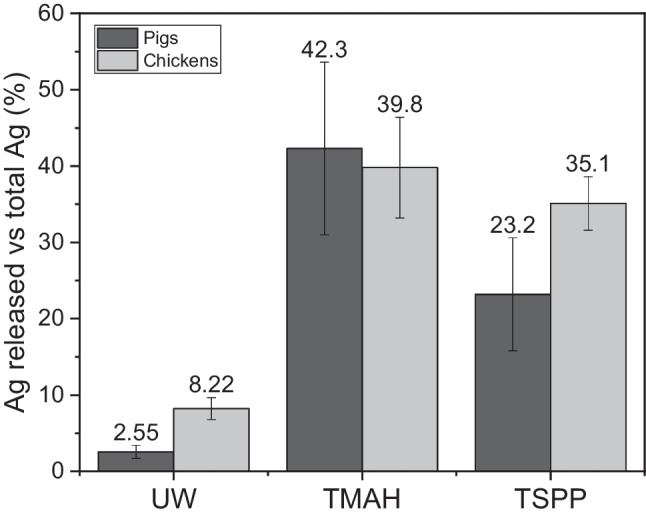
Percentage of total extracted silver from faeces of pigs and chickens using ultrapure water, TMAH with cysteine and Triton-X and TSPP sequential extraction by ICP-MS

**Fig. 2:**
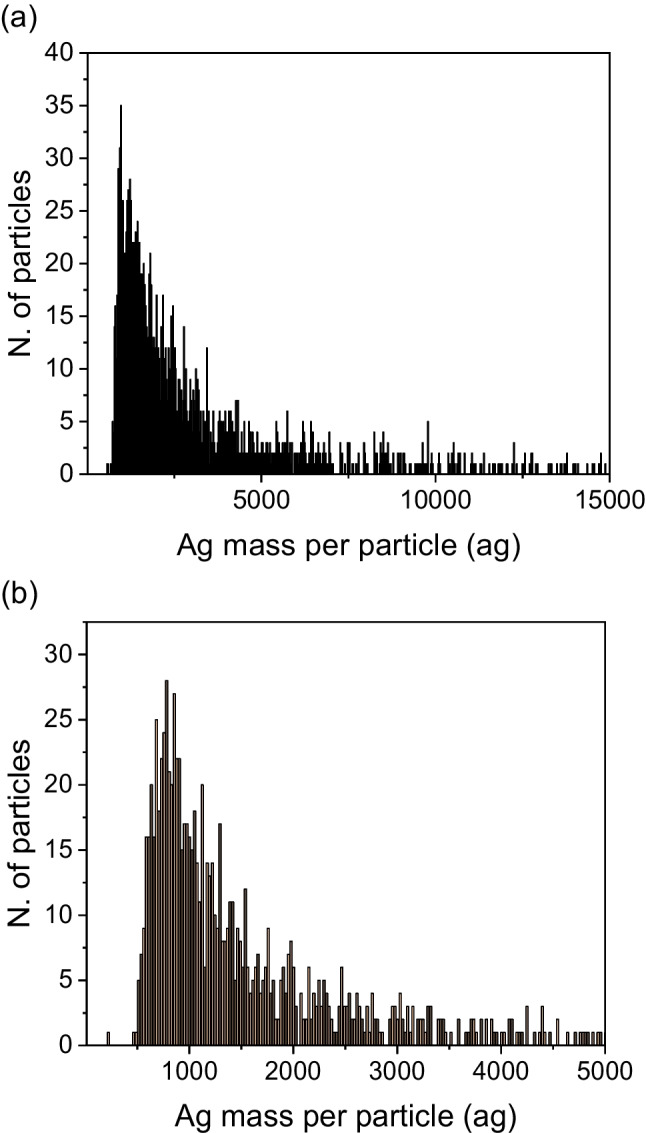
Distribution of Ag-containing particles in the leachates of (**a**) pigs and (**b**) chickens faeces by SP-ICP-MS

Water extraction is considered an approach that evaluates the potential release of substances from waste materials [38]. Figure 1 shows the percentage of total Ag released in each extraction versus the total Ag obtained by the acid digestion, whose mean values are in the samples section. The total Ag released in faeces leachates using ultrapure water, measured by ICP-MS, was 2.5 ± 0.9% for pigs and 8.2 ± 1.4% for chickens. This little percentage of release means that the impact of the faeces in the environment would be low if they were used as fertilizers. The different silver species in the leachates were characterized by different techniques: SP-ICP-MS, HDC-ICP-MS and AF4-ICP-MS. Concerning SP-ICP-MS analysis (Fig. 2), few silver particles were found; between 0.1 and 10% of the total Ag was released in a nanoparticulate form. The detection of the NPs in SP-ICP-MS analysis is limited to the size limit of detection (LOD_size_) [39], which depends on the dissolved species concentration in every sample. When the concentration of the dissolved species increases, so do the baseline and the LOD_size_. The smallest particles will not be detected, and they were included in the baseline; thus, the particle concentration is underestimated [40]. In our samples, because of the high concentration of dissolved silver, not all the particle distribution is seen, and only the tail of the distribution is obtained. In general, the LOD_size_ obtained for the samples was around 35–40 nm, which means that particles with a smallest size could not be quantified as particles but as dissolved form, and their concentration is underquantified. The most frequent mass of silver per particle found was less than 1 fg per particle (0.781 fg for chicken and 0.971 fg for pigs), which is equivalent to a diameter of 34 ± 2 nm for chickens and 35 ± 9 nm for pigs (figure S1 in SI).

**Fig. 3:**
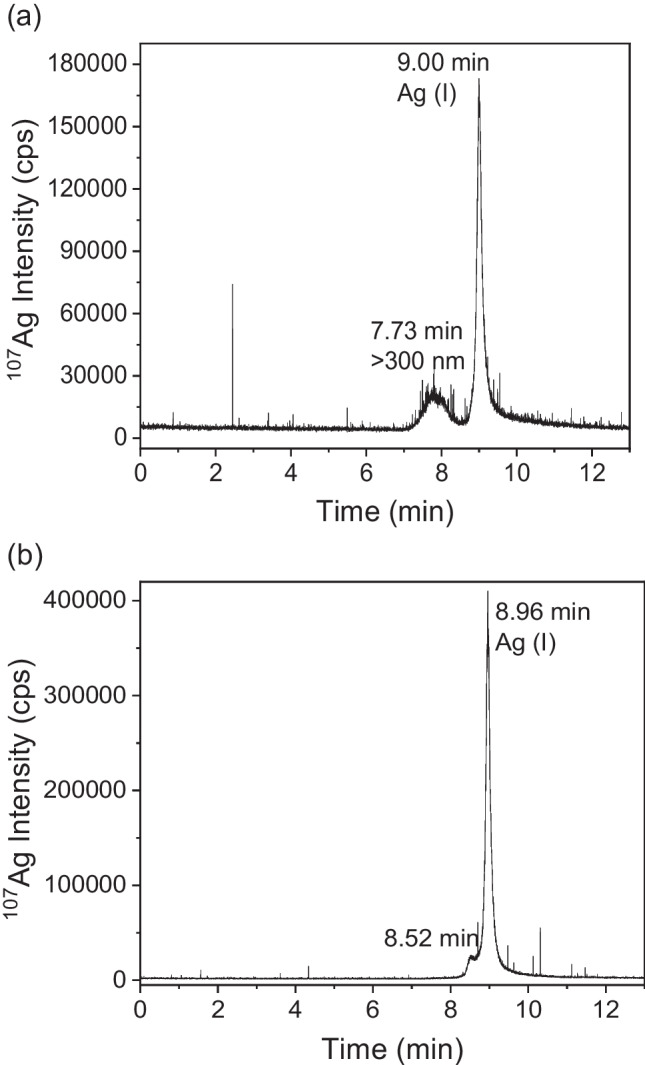
HDC-ICP-MS chromatograms for the aqueous leachates of (**a**) pigs and (**b**) chicken faeces

In Fig. 3, the HDC-ICP-MS chromatograms obtained for the water extraction of pig (Fig. 3a) and for chicken (Fig. 3b) faeces are shown. Two peaks were seen in both chromatograms, one peak eluted at 8.96 ± 0.04 min, which corresponds to Ag(I) (chromatogram of standard of Ag(I) in water is shown in figure S2 in SI) and appears in both animal leachates. For pigs, another peak was observed at 7.73 min corresponding to the elution of Ag-containing particles with sizes larger than 300 nm, which is the size limit of the HDC column that the manufacturer set as the maximum size of separation. For chicken faeces, a second peak appears at 8.52 min in the leachates, which could correspond to another form of Ag(I) complexed to other biomolecules. These findings agree with TEM results (figure S3 in SI), in which particles and agglomerates with sizes between 100 nm and 1 μm were found. With EDS, the composition of some of these complexes included Ag_2_S, while Al and Si observed in the EDS spectrum confirmed the presence of other particles that were bound to kaolin which is the support used in the nanomaterial fed to the animals [41].

The leachates of pig faeces were also measured by AF4-ICP-MS. The results obtained agree with the ones obtained by HDC-ICP-MS (Fig. 3a) and TEM (figure S3 in SI). In the fractogram obtained (figure S4a in SI), there is a small peak at 6.40 min corresponding to dissolved silver forms and another big peak which corresponds to bigger particles. Several dilutions of a sample were analysed to verify the un-alteration of the particles during the process. The comparison of the fractograms resulting from the different dilutions shows that depending on the dilution, the particle retention time changes, shifting into shorter retention times as the dilutions increased (figure S4b in SI). This could mean that the particles size changes depending on the dilutions due to changes in the equilibriums inside the sample. Therefore, water is not a good extractant of the particles in this matrix, due to its inability to maintain the size of the particles and its dependence on the dilutions.

### Alkaline extraction

TMAH extraction was used in the bibliography to extract AgNPs from several biological tissues [11, 12, 22, 24, 26, 42]. TMAH is capable to digest organic and biological matrices due to its ability to break down the disulphide chemical bonds of proteins [12, 22] without changes in the Ag(I) and AgNPs. In the study of Jiménez-Lamana et al. [15], cysteine was added to the solution to stabilize Ag(I) by binding it because of its thiol groups [43, 44]. Triton X-100 is a non-ionic surfactant, and it is added to the extraction solutions to stabilize the small particles due to lower surface tension [11, 45] and to discard the formation of silver particles from ionic silver [22].

In this study, the method used by Jiménez-Lamana et al. [15] was applied. The centrifugation conditions used in [15] were 3000 rpm for 15 min; however, in our study, it was observed that using these conditions, part of silver was retained in the solid. Therefore, centrifugation and sedimentation were compared as separation methods for the alkaline extracts of both animals to study the possible silver losses by ICP-MS. The results are shown in Table 1. With centrifugation, a loss of Ag is observed obtaining an amount of total Ag between 7 and 15%, while with sedimentation, the released silver increased to 30–42%. This agrees with the bibliography [28, 29], in which a loss of Ag in the supernatant is reported. So, sedimentation was chosen as the separation method for the extraction of faeces.

**Table 1:** Effect of the centrifugation, sedimentation and stabilizer on the alkaline extraction efficiency of silver from pig and chicken faeces by ICP-MS

	TMAH, cysteine and Triton		TMAH and CaCl_2_
Samples	Total Ag released (%) with centrifugation	Total Ag released (%) with sedimentation	Total Ag released (%) with sedimentation
Pig faeces	14.9 ± 5.3	42.3 ± 11.3	20.5 ± 6.8
Chicken faeces	9.8 ± 0.6	39.8 ± 6.6	40.4 ± 3.3

The results obtained for the alkaline extraction with the sedimentation process are shown in Fig. 1. For pigs, only 42 ± 11% of the total Ag is extracted. For chickens, the Ag extracted is 40 ± 7%. This low percentage of extraction is caused by remaining residues of the indigested faeces which act like a “trap” for the silver forms, preventing their complete extraction [42]. The alkaline extracts were also analysed by SP-ICP-MS. The transport efficiency was measured in ultrapure water and, also, in the presence of 1% TMAH. The two gold calibration curves have shown a difference between the slopes applying the student *t* test; consequently, the transport efficiency used was calculated using 1% TMAH. The calculated transport efficiency values were slightly different giving 7.22 ± 0.03% for water calibration and 8.63 ± 0.10% for TMAH calibration. The percentage of the particles released was lower than 1% regarding the total Ag released (the mass distributions are shown in figure S5 in SI). This percentage represents a very low concentration of particles, which was below the limit of detection of the HDC-ICP-MS (17.0 μg L^−1^). That is the reason why in the chromatogram obtained for HDC-ICP-MS for pigs (see figure S6 in SI), only one peak appears at 8.71 min, which corresponded to Ag (I).

Due to the possibility of the precipitation of Ag(I), Clark et al. [23] used a solution of CaCl_2_ for the extraction of Ag(I) and AgNPs in fish tissues to avoid the formation of particles of Ag from Ag(I). In our case, the use of CaCl_2_ as a stabilizer to extract silver species from faeces was also evaluated and compared to the use of cysteine and Triton. The results are shown in Table 1. For chickens, there was no difference between both extraction stabilizers; however, for pigs, the extraction with CaCl_2_ released less Ag. Thus, the nature of the matrix and the initial quantity of silver in the real sample might affect the extraction efficiency. In the distributions obtained by SP-ICP-MS, chicken faeces had similar distributions for both stabilizers (figure S7b in SI) while distributions for pigs (figure S7a in SI) were different. Extraction with CaCl_2_ gave smaller particles than cysteine extraction and a smaller number of particles extracted using the same dilution. The low efficiency of extraction using CaCl_2_ in pig faeces did not allow detecting enough particles.

### TSPP sequential extraction

**Fig. 4:**
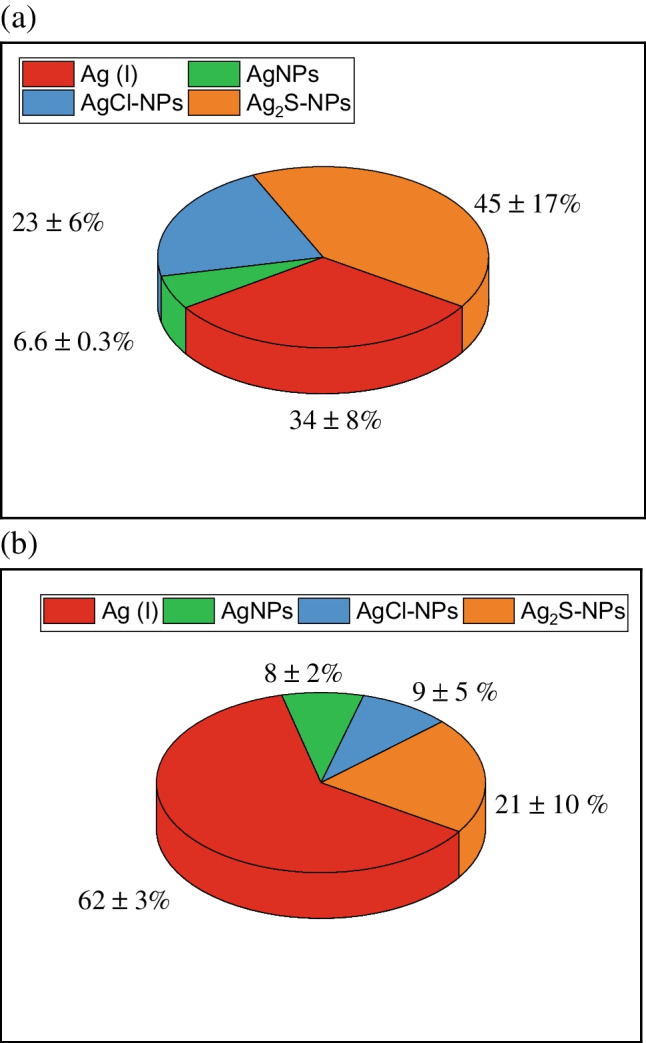
Percentage of silver forms in faeces of (**a**) pigs and (**b**) chickens using the TSPP scheme of fractionation

To obtain the different fractions of silver species, a sequential extraction developed by Hong et al. [30] for spiked soils was studied. In this scheme of fractionation, TSPP, NH_3_ and Na_2_S were used to separate the different fractions of the Ag: Ag(I), AgNPs, AgCl-NPs and Ag_2_S-NPs. The total % of Ag released is shown in Figs. 1 and 4, and the percentage of each species of Ag is presented considering the Ag extracted by the sequential extraction as being the total Ag extracted (100%) (concentration of Ag species released in table T3 in SI). There were differences in the predominant species depending on the animal. For chickens (Fig. 4b), most of Ag was in Ag(I) form (62%), like the results found with alkaline extraction. The rest of the Ag was present in the form of Ag_2_S-NPs (21%), AgNPs (8%) and AgCl-NPs (9%). For pigs (Fig. 4a), most of the Ag extracted was in Ag_2_S-NP form (44%). Ag(I) represents 34% of the total Ag extracted. The rest of the Ag was in AgNPs form and only 2% of the Ag was extracted as AgCl-NPs. The extraction efficiency obtained by Hong et al. was between 70 and 110% of the total Ag spiked in the soils studied, whereas in this study, the extraction efficiency (Fig. 1) was 23 and 35% for pig and chicken faeces, respectively. These low extraction values can be explained by the fact that the method was developed to extract Ag species in spiked soils, but in our case, the Ag species were already in the faeces; thus, the difference in the matrix (organic matter, pH) might affect the level of extraction. The advantage of the TSPP scheme is the isolation of different fraction and the qualitative characterization of the nature of particles (Ag_2_S-NPs, AgNPs, AgCl-NPs). With the sequential extraction, the total Ag released was lower than the amount released by alkaline extraction (see Fig. 1). This is because TMAH is capable to digest the organic matter better than the TSPP and Na_2_S, resulting in a higher release of the organic complex of Ag [28]. Nevertheless, this sequential extraction was useful to know the different forms of Ag.

### Behaviour of the different Ag species through the extraction methods

To study the behaviour of Ag species during the various extraction methods employed and to understand the effects of matrix for the different type of samples, the extractions of spiked control samples were carried out using all the mentioned protocols.

#### Water extraction

Control faeces samples were spiked with Ag(I) and 40 nm AgNPs and then were subjected to the protocol of water extraction. ICP-MS results obtained for total silver determination (figure S8 in SI) are different for pigs and chickens. Meanwhile, for chickens, values were 80–88% for Ag(I) and AgNP spiked faeces; for pigs, the recovery for Ag(I) spiked solution was 1.8 ± 0.7%, and for AgNPs spiked solution was 24.8 ± 1.9%. This difference can be caused by the different compositions of the animal faeces. Moreover, the total Ag released was higher for chickens than for pigs (Fig. 1). The recoveries obtained in the spiked samples agree with that difference. The recoveries obtained by SP-ICP-MS confirm these results. While for chickens, the recovery for Ag(I) was more than 90%, and for AgNPs, more than 77%; for pigs, the recovery was very low (almost 4% for Ag(I) and 23% for AgNPs (see table T4 in SI). There were no particles in the Ag(I) spiked solution for both animals. This indicates that there was no precipitation of Ag(I) during the leaching process. In the AgNP spiked solution for pigs (size distributions in figure S9a in SI), the sizes of the particles found were smaller (28.9 ± 2.5 nm) than the original size (40 nm). This means that the Ag particles oxidize and break during this process, which is reported in the bibliography [31], so it cannot be ensured that the particles which appear in the faeces leaching (Fig. 2a) are the original particles, or they have suffered changes during the process. However, for chickens (figure S9b in SI), the particles found had the same mean size than the original NPs spiked (40.5 ± 0.07 nm), which was also observed in other studies where the equivalent particle sizes of Ag-containing particles extracted from soils with ultrapure water were consistent with the original sizes of the spiked AgNPs sizes [28, 29]. However, although the size was consistent with the AgNPs initially added to the chicken faeces, it is doubtful that the particles remained untransformed during the procedure; thus, secondary precipitates such as the formation of Ag deposited onto organic or clay surfaces, Ag_2_S and AgCl may be detected also. The difference in the matrix for both animals is behind this dissimilarity in the behaviour of the spiked AgNPs. These differences in recovery values were also observed for spiked soils. Whereas Torrent et al. [31] reported recovery values of less than 8% for Ag(I), a small fraction of the AgNPs, Mahdi et al. [7] obtained 39% recovery for the extraction of AgNPs from soils using ultrapure water.

**Fig. 5:**
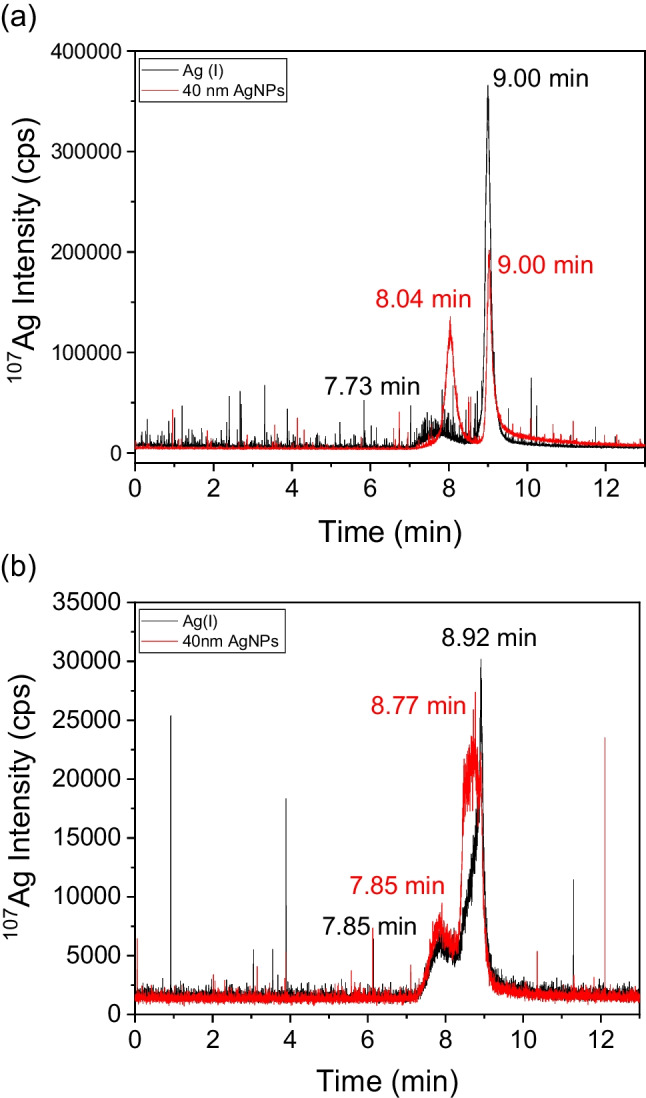
HDC-ICP-MS chromatograms (**a**) spike of Ag(I) and 40-nm AgNPs over control pigs faeces (**b**) spike of Ag(I) and 40-nm AgNPs over control chickens faeces

The spiked control faeces after water extraction were also analysed by HDC-ICP-MS. In the chromatograms obtained for the Ag (I) spiked solution (Fig. 5, black line), two peaks appear for pigs (Fig. 5a) and for chickens (Fig. 5b), one of which corresponds to Ag(I), at 9.00 min for pigs and 8.92 min for chickens, and another small peak appears at 7.73 min for pigs and 7.85 min for chickens, which would correspond to Ag bound to kaolin particles, giving sizes greater than 300 nm. This chromatogram was similar to the chromatogram obtained in the original Ag samples; therefore, it was not possible to confirm the presence of AgNPs in the sample, whereas the presence of Ag bound to kaolin particles or Ag aggregates was confirmed.

Regarding the spike of AgNPs (Fig. 5, red line), two peaks appeared, but they were different for both animals. For pigs (Fig. 5a), one of the peaks corresponded to particles around 60 nm, with a retention time of 8.04 min, and another peak at 9.03 min, corresponding to Ag(I). For chickens (Fig. 5b), one of the peaks was seen at a retention time (8.77 min) close to the retention time of Ag(I) and a previous peak appeared at 7.85 min, the same that was obtained in Ag(I) spiked solution. With these results, the oxidation of the AgNPs in the leaching for both animals would be confirmed, which agrees with what was found by SP-ICP-MS.

#### Alkaline extraction

For the study of the behaviour of silver during the alkaline extraction, spiked control samples with Ag(I) and 40 nm AgNPs were subjected to this extraction. In the comparison of centrifugation and sedimentation for the alkaline extraction of the spiked faeces, a loss of Ag is observed as in samples. For the spike of Ag(I) in pig faeces, 41.3 ± 7.2% is recovered with centrifugation versus 77.3 ± 7.2% recovered with sedimentation. For the spike of AgNPs, the difference is lower, 54.5 ± 1.2% with centrifugation and 64.8 ± 2.6% with sedimentation. Same effect for chickens was obtained, although the difference between centrifugation and sedimentation was less than for pigs. The obtained recoveries were 85.0 ± 0.8% for the Ag(I) spiked sample and 77 ± 7% for the AgNPs spiked sample. The results confirm that centrifugation affects the recovery of Ag as in samples [28, 29]. The differences in the composition of the faeces of both animals could explain the differences in the recoveries.

The analysis of alkaline extraction by SP-ICP-MS confirmed the presence of particles in the Ag(I) spiked control samples which means that a fraction of the Ag(I) was precipitating during alkaline extraction (figure S10 in SI). Almost 4% of Ag was recovered by SP-ICP-MS in its precipitated form. Vidmar et al. [18] explained that the precipitation of Ag in samples is caused not only because of the presence of TMAH but also because of the components of the samples, specifically, –OH groups of the proteins present in faeces, which can create an ideal atmosphere for the formation of particles. So, it can be concluded that the precipitation of the Ag(I) present in the faeces contributed to the appearance of Ag particles in the samples.

For the AgNP spiked samples, the size obtained for both animal faeces (40 ± 1 nm) was similar to the original size of the AgNPs (40 nm), and no dissolution was observed. A small shift of the distributions to bigger particle sizes and a decrease in NP frequency was seen (figure S11 in SI) using the particle distribution in ultrapure water as a reference. In the literature, Vidmar et al. [18] observed a slight shift of AgNPs in placental tissue to a larger size using the TMAH extraction; however, the author stated that TMAH alone does not lead to particle formation in the absence of biological matrix; besides, the tissue spiking gave similar size distribution of that of pristine AgNPs. Then, alkaline extraction is a useful extraction method since it does not change the size of the particles which agrees with what is said in the bibliography [11, 22–24], but it is not a suitable extraction method for Ag(I) due to the precipitation observed in the Ag(I) spiked solutions.

#### TSPP sequential extraction

For the sequential extraction, Ag(I) and 40-nm AgNPs were spiked to control faeces and subjected to the TSPP scheme of fractionation. The recoveries are shown in Table 2. The total Ag recovered was about 80% of the Ag spiked for both suspensions, which is consistent with the article of Hong et al. [30] in which the total Ag and AgNPs spiked in the soil were recovered. In theory, this extraction should mobilise all the Ag in the samples, but faeces subjected to the reported scheme released less than 30% of Ag. Bindings between the different forms of silver and the matrix are stronger in the real sample than when the control sample is spiked, so, for the analysis of an extraction protocol, it is necessary, not only the study of spiked samples because the behaviour could be different from real samples. Other authors found TSPP extraction to be the optimal extraction method for AgNPs; however, it gave lower Ag (I) concentration released, while 25% TMAH gave similar AgNP extraction to TSPP with higher amount of Ag(I) due to more digestion of the solid organic matter [28]. Contrarily, Li et al. [46] also compared the use of ultrapure water, NaNO_3_, KNO_3_ and TSPP solutions for the extraction of AgNPs from soils and sediments; the optimal method was TSPP extraction. However, we must outline that the samples used in the mentioned studies were spiked soils, while in our study, the samples are faeces collected from intestines of the animals that were orally administrated a silver-kaolin nanomaterial. In our work, low percentages of recovery are obtained.

**Table 2:** Silver recovery (%) of spiked samples of pig and chicken faeces extracted with TSPP sequential extraction by ICP-MS

Animal	Sample	Ag recovery (%)
Pig	Control faeces spiked with Ag(I)	77.4 ± 1.4
	Control faeces spiked with 40 nm AgNPs	88.3 ± 11.0
Chicken	Control faeces spiked with Ag(I)	95.5 ± 3.4
	Control faeces spiked with 40 nm AgNPs	78.2 ± 5.3

### Comparison between the extraction methods

Overall, the comparison of the extraction methods employed throughout this study permitted to conclude that the use of TMAH extraction combined with SP-ICP-MS analysis is useful to characterize the silver-containing particles in faeces. For the AgNPs spiked samples, no transformation or oxidation of the NPs was confirmed. Nevertheless, Ag-containing particles were found in the Ag (I) spiked samples, so we cannot ensure that all the particles which appear in the SP-ICP-MS distribution correspond to Ag particles. Moreover, it can be concluded that the faeces matrix (pig or chickens) in real samples affects the extraction of AgNPs because for spiked control samples, the recoveries were in the range of 65–78%, whereas in real samples were much lower (around 40%).

Although the TSPP method gave lower extraction efficiencies than TMAH method in real samples, it enabled to fractionate the main silver forms in the sample from a qualitative point of view. Different silver species have been found, such as AgCl-NPs and Ag_2_S-NPs, in which it is not possible with TMAH method. It is important to underline that the silver species do not behave in the same way when they are in a real sample or when they are spiked on a control sample.

The low percentage of the Ag mobilized made the water extraction a poor quantitative extraction method. Besides, because of the changes suffered by Ag(I) and the AgNPs during the leaching process, it is impossible to ensure the fraction of the silver particles and Ag(I) present in the faeces, as well as to identify the form of Ag. For this reason, water extraction is not a convenient approach for the extraction of silver from faeces, although it is a good option to evaluate the release of total silver from faeces and to determine its subsequent fate and exposure for environmental risk characterization.

The level of uncertainty associated to three extraction methods was evaluated through the repeatability. The repeatability for water extraction was in the range of 6.2–7.6 % (*n* = 3) being similar as for the alkaline extraction with TMAH, cysteine and Triton (between 0.7 and 6.0 % (*n* = 3)). Meanwhile, for the sequential extraction, the repeatability was in the range of 1.8–17% (*n* = 3). The higher values obtained for the sequential extraction are because of all the steps needed in this extraction, which makes the accumulation of errors and causes a decrease into the repeatability.

## Conclusions

The comparison of several extraction methods reported in the bibliography was conducted to demonstrate the applicability of these procedures for the extraction of silver species present in the faeces of pigs and chickens fed with a silver-based nanomaterial. Water extraction is typically used to simulate the release of chemicals from amendments and wastes into the environment. In this study, water extraction resulted in the release of less than 10% of the silver present in pig and chicken faeces, with ionic silver as the predominant form of silver found. The observed changes of silver species during the leaching process make water extraction not a convenient method for the extraction of silver from faeces. Alkaline extraction by using TMAH allows the digestion of organic matter and the extraction of intact silver-containing particles. However, this study has shown that a fraction of the Ag(I) was precipitated during alkaline extraction contributing to the appearance of Ag particles in the samples. TSPP extraction allowed the fractionation of silver into Ag (I) and three particulate fractions, namely, AgNPs, AgCl-NPs and Ag_2_S-NPs, which cannot be differentiated by any other extraction method. The relevance of analysing real samples instead of spiked samples has become evident because the silver species extracted did not behave in the same way. In all cases, a small fraction of silver-containing particles was detected. Although each extraction procedure allowed extracting specific silver species, none of these protocols gave a high extraction efficiency to recover all the silver forms in the original samples. These findings reveal the difficulty of characterizing species derived from nanomaterials in real complex samples such as faeces.

## Supplementary information

ESM 1:
