# Supplementary material for: Comparative study of extraction methods of silver species from faeces of animals fed with silver-based nanomaterials
Source: Mikrochim Acta. 2023 May 9;190(6):204. doi: 10.1007/s00604-023-05777-0 (PMC10169895; doi:10.1007/s00604-023-05777-0)
Supplement: Supplementary file 1 — ESM 1: [file 604_2023_5777_MOESM1_ESM.docx]

**Comparative study of extraction methods of silver species from faeces of animals fed with silver-based nanomaterials**

Mariam Bakir, Khaoula Ben-Jeddou, María S. Jiménez ^*^, Eduardo Bolea, Josefina Pérez-Arantegui, Francisco Laborda.

Group of Analytical Spectroscopy and Sensors (GEAS), Institute of Environmental Sciences (IUCA), University of Zaragoza, Pedro Cerbuna 12, 50009 Zaragoza, Spain.

**Supporting information**

**Table T1.** Instrumental and data acquisition parameters for ICP-MS in conventional mode, SP-ICP-MS, HDC-ICP-MS and AF4-ICP-MS.

| Instrumental parameters for NexION 2000 ICP-MS | | |
| --- | --- | --- |
| RF power | 1600 W | |
| Argon plasma flow rate |  | |
| Plasma | 15 L min^-1^ | |
| Auxiliary | 1.2 L min^-1^ | |
| Nebulizer | 1.04 L min^-1^ | |
| **ICP-MS** |  | |
| Dwell time | 50 ms | |
| Replicas | 10 | |
| Settling time | 0 ms | |
| Isotopes monitored | ^107^Ag, ^109^Ag | |
| **SP-ICP-MS** | 120 | |
| Dwell time | 100 µs | |
| Total acquisition time | 50 s | |
| Isotopes monitored | ^107^Ag | |
| Instrumental parameters for ELAN DRC-e ICP-MS | | |
| **ICP-MS** | |  |
| RF power | | 1100 W |
| Argon plasma flow rate | |  |
| Plasma | | 15 L min^-1^ |
| Auxiliary | | 1.2 L min^-1^ |
| Nebulizer | | 1.0 L min^-1^ |
| Sweeps per reading | | 1 |
| Dwell time | | 50 ms |
| Acquisition mode | | Peak hoping |
| Isotopes monitored | | ^107^Ag, ^109^Ag |
| **HDC** | | 120 |
| Column | | PL-PSDA type 1 |
| Flow rate | | 1.6 mL min^-1^ |
| Injection volume | | 50 µL |
| **AF4** | |  |
| Loop volume | | 100 µL |
| Flow rate | | 1 mL min^-1^ |

**Table T2.** Crossflow methods used AF4 separation

| **Program step** | **Time (min)** |  | **Crossflow (mL min^-1^)** |
| --- | --- | --- | --- |
| **Program A:** |  |  |  |
| **Injection/focusing** | 5 | (Injection Flow, 0.20 mL min^-1^) | 4 |
| **Separation** | 20 | Constant | 3 |
|  | 2 | Linear decay | 3 to 0 |
|  | 5 | Constant | 0 |
| **Program B** |  |  |  |
| **Injection/focusing** | 4 | (Injection Flow, 0.20 mL min^-1^) | 3 |
| **Separation** | 5 | Constant | 3 |
|  | 10 | Linear decay | 3 to 0 |
|  | 1 | Constant | 0 |

**Figure S1.** Size distributions of Ag-containing particles in leaching of (a) pig and (b) chicken faeces by SP-ICP-MS.

**Figure S2.** Chromatogram of standard of Ag (I) in ultrapure water.

(a)


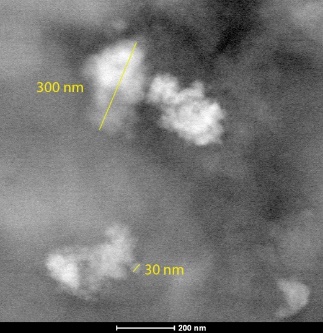

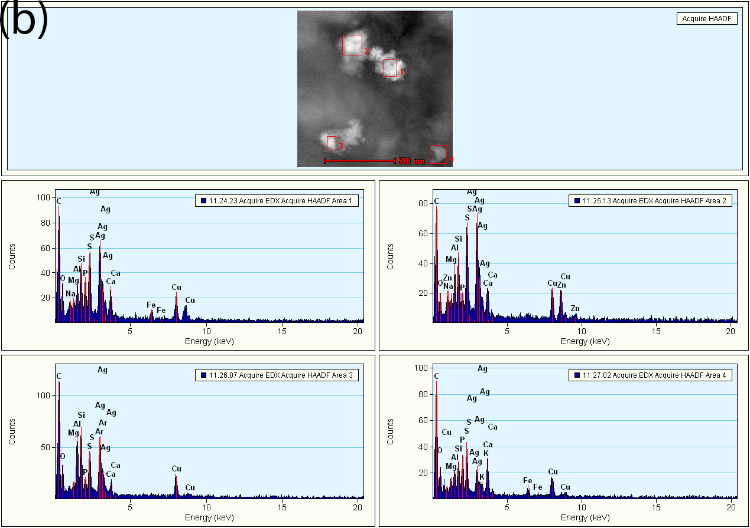


b)


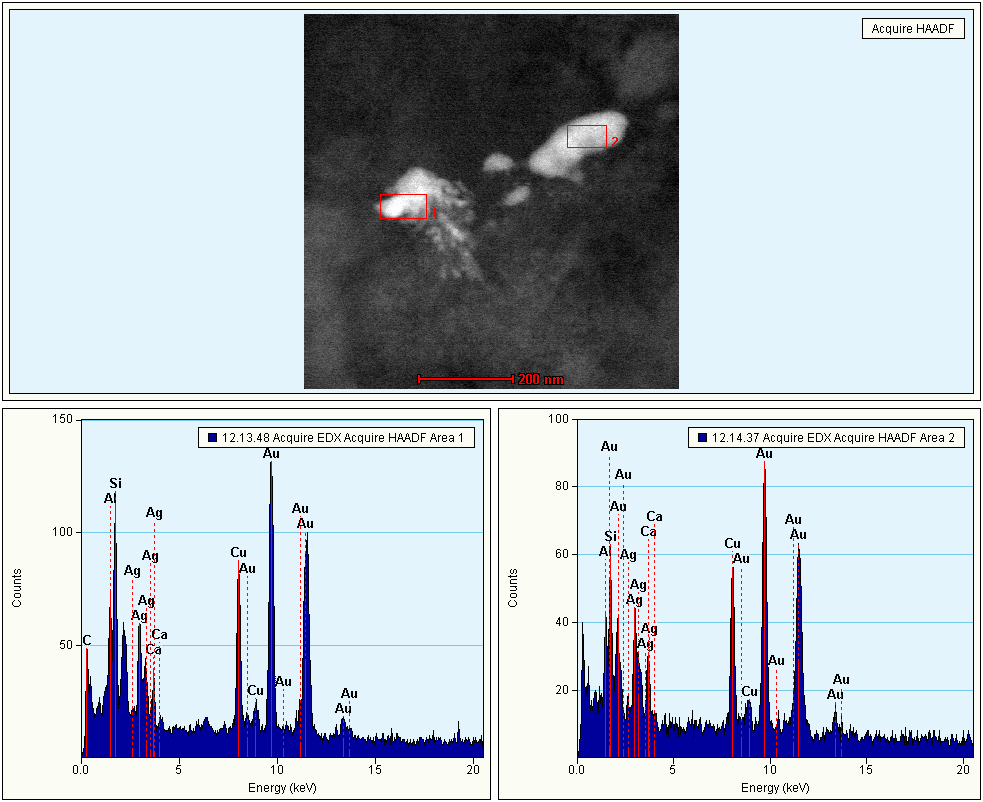


**Figure S3.** (a) TEM images and EDS spectrums of Ag-containing particles in leachates of (a): pig and (b): chicken faeces

**Figure S4.** AF4-ICP-MS fractograms for pig faeces leachates. (a) Fractogram for leachates without dilution. (b) Fractogram of different dilutions of leachates, (without dilutions and 1:2, 1:4, 1:6 and 1:10 dilutions).

**Figure S5.** Distribution of Ag-containing particles in alkaline extracts of (a) pig and (b) chicken faeces by SP-ICP-MS

**Figure S6.** HDC-ICP-MS chromatogram of the alkaline extract of pig faeces

**Figure S7.** Ag mass distribution of silver-containing particles in (a) pig and (b) chicken faeces extracts using two stabilizers and TMAH.

**Table T3.** Concentration of Ag (mg kg^-1^) in pig and chicken faeces by ICP-MS after TSPP and Na_2_S extractions (values are the median of two samples)

| Animal | | Total Ag extracted  (mg kg^-1^) | Ag (I) extracted  (mg kg^-1^) | Ag_2_S-NPs extracted  (mg kg^-1^) | AgNPs extracted  (mg kg ^-1^) | AgCl-NPs extracted  (mg kg^-1^) |
| --- | --- | --- | --- | --- | --- | --- |
| Pigs | **Ag20** | 31.7 ± 7.9 | 9.5 ± 1.4 | 4.2 ± 1.3 | 0.2 ± 0.1 | 18.0 ± 10.6 |
|  | **Ag200** | 269.0 ± 13.0 | 99.4 ± 13.1 | 75.2 ± 28.4 | 18.3 ± 0.1 | 94.4 ± 28.4 |
| Chickens | | 26.2 ± 2.6 | 16.3 ± 0.7 | 5.8 ± 3.3 | 1.9 ± 0.3 | 2.2 ± 1.0 |

**Figure S8.** Recoveries of spiked control faeces for the leaching by ICP-MS.

**Table T4.** Particles size and recoveries of the leachates of Ag (I) and 40 nm AgNPs spiked over the control faeces (%) by SP-ICP-MS.

| Sample | Pigs | | Chickens | |
| --- | --- | --- | --- | --- |
|  | **Size (nm)** | **Recovery (%)** | **Size (nm)** | **Recovery (%)** |
| Control faeces spiked with Ag (I) | - | 3.8 ± 1.5 | - | 91.0 ± 7.0 |
| Control faeces spiked with 40 nm AgNPs | 28.9 ± 2.5 | 23.3 ± 3.3 | 40.5 ± 0.1 | 77.4 ± 7.0 |

**Figure S9.** Distributions of 40 nm AgNPs spiked over control faeces of (a) pigs and (b) chickens using water extraction by SP-ICP-MS

**Figure S10.** Distribution of Ag-containing particles in the TMAH extracts of the spike of Ag (I) into control faeces of (a) pigs and (b) chickens by SP-ICP-MS.

**Figure S11.** Distribution of 40 nm AgNPs in water and in spiked faeces extracted by TMAH-Cysteine-Triton extraction (a) pigs and (b) chickens by SP-ICP-MS.
